# Oral Health in Alzheimer's Disease: A Life‐Course Perspective on Clinical Management and Caregiver Support

**DOI:** 10.1111/scd.70179

**Published:** 2026-05-07

**Authors:** Mayron Guedes Silva, Matheus Bastos Vasconcelos, Raphael Guedes Silva, Maria Áurea Lira Feitosa

**Affiliations:** ^1^ Dentistry Graduate Program Federal University of Maranhão (UFMA) São Luís Maranhão Brazil; ^2^ Health Sciences Graduate Program Federal University of Maranhão (UFMA) São Luís Maranhão Brazil; ^3^ Department of Dentistry Federal University of Maranhão (UFMA) São Luís Maranhão Brazil

**Keywords:** aged, Alzheimer disease, caregivers, dementia, dental care, oral health

## Abstract

Aim: To synthesize current evidence on oral health conditions in individuals with AD and to discuss stage‐specific dental management strategies, including caregiver involvement, from a life‐course perspective. Methods: A comprehensive literature search was conducted in PubMed/MEDLINE, Scopus, Scielo, and Web of Science using MeSH and free‐text terms related to Alzheimer's disease, oral health, and caregivers, with no language or time restrictions. Results: Individuals with AD commonly present with periodontal disease, root caries, xerostomia, traumatic mucosal lesions, and prosthesis‐related complications. The available evidence does not strongly support a bidirectional causal relationship; instead, poor oral health and AD appear to be cumulative conditions influenced by shared antecedent factors, such as biological aging and chronic inflammation. Functional dependence and behavioral symptoms further contribute to oral deterioration over time. Effective dental care therefore requires stage‐adapted approaches, ranging from preventive and restorative interventions in early stages to palliative strategies focused on infection control and comfort in advanced disease. Conclusion: Oral health care for individuals with AD should be grounded in a life‐course and biopsychosocial framework, prioritizing realistic, stage‐specific clinical management and structured caregiver support within interdisciplinary geriatric care.

## 1 Introduction

Population ageing has been accompanied by an increased prevalence of chronic neurodegenerative diseases, particularly Alzheimer's disease (AD), the leading cause of dementia worldwide [1]. AD affects approximately 5% of individuals aged 65–74 years and up to 33.4% of those older than 85 years [2]. The disease is characterized by progressive and irreversible cognitive decline, executive dysfunction, and behavioral changes, which substantially impair autonomy and the ability to perform daily self‐care activities [3]. As AD progresses, individuals become increasingly dependent on caregivers, including family members and healthcare professionals [4, 5]. Globally, more than 55 million people currently live with dementia, a number projected to reach 139 million by 2050 [6, 7].

In this context, oral health deteriorates rapidly. Evidence suggests that AD and oral diseases (specifically periodontitis and caries) are cumulative conditions that share common antecedent exposures, such as biological aging, chronic systemic inflammation, and social determinants of health [8, 9, 10, 11, 12]. Rather than a simple causal link, a syndemic burden is observed: cognitive deficits and motor loss lead to poor oral hygiene, while medication‐induced xerostomia accelerates dental destruction [13, 14]. This creates a scenario of generalized oral health collapse, where the progressive loss of dental and mucosal integrity mirrors the patient's functional decline.

Dental care for individuals with AD presents substantial clinical challenges, including reduced cooperation, communication barriers, altered pain perception, and ethical complexities in treatment planning, especially in advanced stages of disease [15]. Access to care is further limited by functional dependence, institutionalization, and a shortage of professionals trained in geriatric dentistry and dementia care [16], highlighting the need for adapted, patient‐centered clinical strategies.

Within this framework, caregivers play a central role in maintaining oral health in older adults with AD [4]. They are generally responsible for daily oral hygiene practices, monitoring oral conditions, scheduling dental appointments, and facilitating communication with healthcare providers. Despite their importance, caregivers often receive insufficient guidance and training in oral healthcare, which may lead to inadequate practices and an increased physical, emotional, and occupational burden [4, 17]. Therefore, the effective integration of both family and professional caregivers into the planning and delivery of dental care is essential to improve oral health outcomes in this population.

Although the body of literature addressing oral health in individuals with AD has expanded in recent years, the available evidence remains fragmented, and practical clinical recommendations for dental professionals are not always presented in a systematic manner. Accordingly, the aim of this comprehensive narrative review is to synthesize current evidence on oral health conditions in AD and to discuss stage‐specific dental management strategies, emphasizing the integration of caregivers into the interdisciplinary care team.

## 2 Methods

A comprehensive narrative review was conducted following the framework proposed by Green et al. [18] to synthesize current evidence on dental care for older adults with AD. This approach enabled the integration of heterogeneous evidence, including observational and qualitative studies, clinical reports, and review articles [19, 20] providing a clinically oriented perspective on care challenges in this population.

### 2.1 Information Source and Search Strategy

Electronic searches were performed in November 2025 in the PubMed/MEDLINE, Scopus, Scielo, and Web of Science databases. The search strategy combined MeSH terms and free‐text keywords related to AD and oral health, using Boolean operators (AND, OR) and truncation. Key terms included (“*alzheimer's disease*” OR alzheimer*) AND (“*elderly*” OR “*older people*” OR “*older adults*”) AND (“*dental care*” OR “*oral health*”). A complementary search incorporated the term “caregiver*” to identify studies addressing caregiver involvement. No restrictions were applied regarding year or language of publication.

### 2.2 Eligibility Criteria

Studies were included if they addressed oral health conditions, dental management, preventive or therapeutic dental care, or caregiver roles for older adults with AD. Excluded articles focused solely on other dementias, pharmacological or neurological topics, or laboratory research. Editorials, letters, and opinion pieces without clinical relevance were also excluded.

### 2.3 Selection Process and Data Screening

Two reviewers (M.G.S. and M.B.V.) independently screened titles and abstracts according to the eligibility criteria. Potentially eligible studies were assessed in full text. Disagreements were resolved by consensus or consultation with a third reviewer (M.A.L.F.). Information on authors, journals, and conflicts of interest was concealed during screening. Reference lists of included studies were also screened.

### 2.4 Data Synthesis

Extracted information was synthesized qualitatively and organized into three thematic domains: (1) Clinical manifestations (caries, periodontitis, lesions); (2) Adapted dental management strategies; and (3) The pivotal role of caregivers.

## 3 Results

The search identified 640 records (MEDLINE = 238; Web of Science = 241; Scopus = 157; SciELO = 4). After duplicate removal (*n* = 203), records were screened according to the eligibility criteria. Twelve studies met the criteria, and manual searches identified 15 additional studies, resulting in 27 included studies.

These included studies, published between 1997 and 2025, comprised observational studies (*n* = 7), literature reviews (*n* = 7), systematic or scoping reviews (*n* = 6), non‐randomized interventional or pilot studies (*n* = 4), randomized controlled trials (*n* = 2), and experimental study (*n* = 1).

### 3.1 AD and Oral Health

**FIGURE 1:**
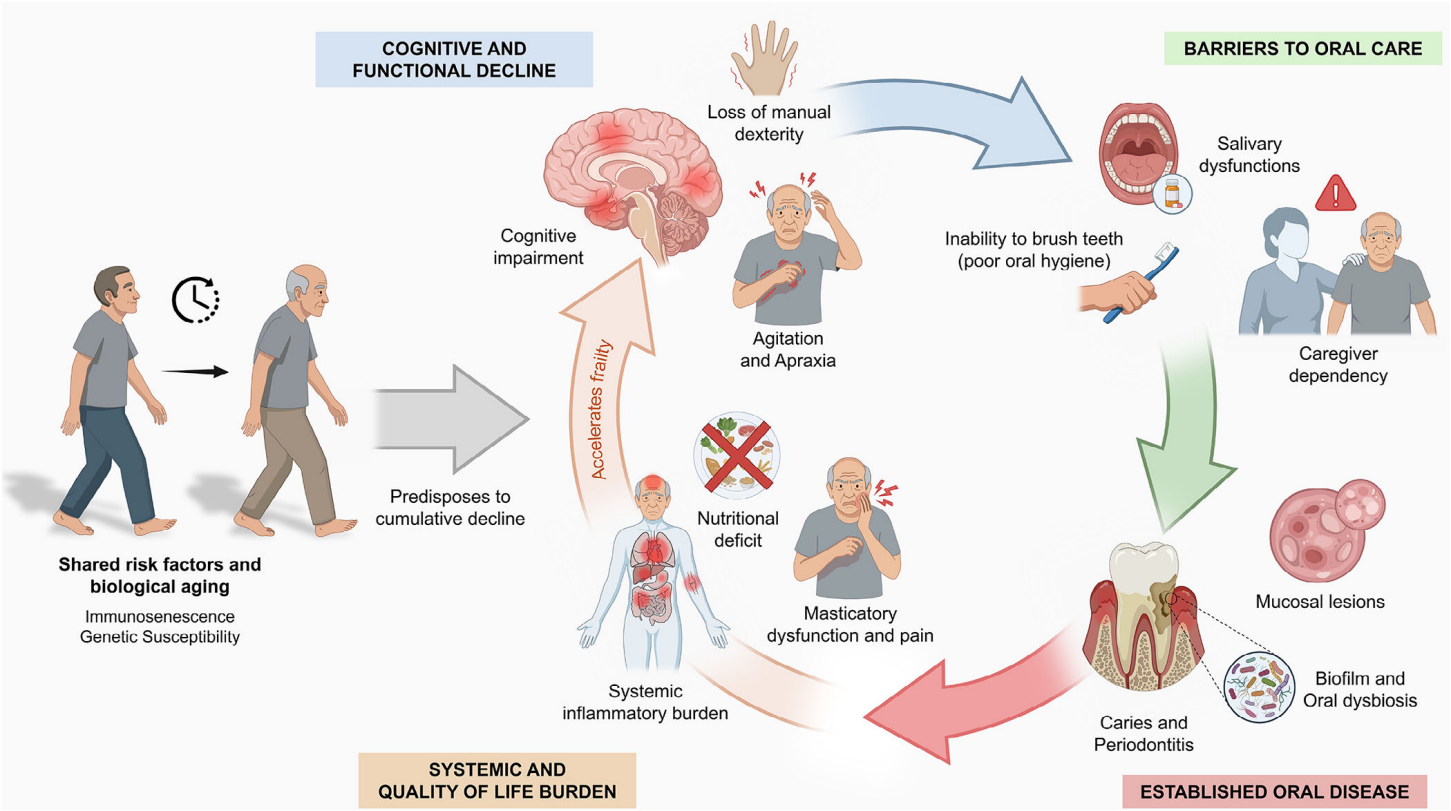
Synergistic cycle of oral and systemic deterioration in Alzheimer's disease. Biological aging, immunosenescence, and shared risk factors predispose individuals to neurodegeneration and oral dysbiosis. (Blue arrow) Alzheimer's disease progression leads to cognitive and functional decline. (Green arrow) Reduced autonomy and medication‐related xerostomia impair oral self‐care. (Red arrow) Oral conditions, such as periodontitis and caries, develop. (Orange arrow) chronic pain, masticatory dysfunctions, nutritional compromise, and systemic inflammatory burden reinforce frailty and overall decline.

Current evidence indicates that AD and oral diseases are co‐occurring conditions that share common risk factors, such as aging, chronic systemic inflammation, and social determinants of health [9, 10, 11, 12]. Rather than a linear causal relationship, a syndemic burden is observed: the cognitive, functional, and behavioral decline associated with AD critically impairs the ability to maintain oral hygiene [10, 15]. This leads to the accumulation of dental biofilm and chronic infection, creating a cycle of generalized health deterioration [10] (Figure 1).

#### 3.1.1 Periodontitis

Periodontitis in individuals with AD represents a significant source of chronic systemic inflammation [11, 12]. Although experimental studies suggest that periodontal pathogens may contribute to neuroinflammatory processes [21, 22, 23, 24], epidemiological evidence remains insufficient to support a bidirectional association. Instead, the coexistence of periodontitis and AD is more plausibly explained by cumulative aging‐related processes, progressive functional decline, and shared long‐term exposures across the life course [9, 10, 25].

Mechanistic hypotheses have investigated the potential role of specific periodontal pathogens, such as *Porphyromonas gingivalis* and *Fusobacterium nucleatum* [21, 22, 23, 24]. These microorganisms are consistently associated with increased systemic inflammatory burden and the production of endotoxins, including lipopolysaccharides, capable of triggering exacerbated inflammatory responses [23, 24, 26]. Experimental evidence suggests the potential translocation of these microorganisms or their components to the central nervous system, where they may contribute to microglial activation, oxidative stress, and the amplification of neuroinflammation [21, 26, 27, 28]. However, establishing definitive causality between oral infections and chronic cognitive disorders such as AD remains challenging, largely due to shared risk factors—including lifestyle, genetic susceptibility, and environmental exposures—that influence both conditions over the life course [29].

As AD progresses, declining manual dexterity, cognitive impairment, and immunosenescence compromise effective biofilm control, resulting in greater plaque accumulation and gingival bleeding compared with cognitively healthy older adults [30]. These observations highlight the need for targeted preventive and therapeutic strategies, including adapted periodontal maintenance protocols, early interventions, and the active involvement of caregivers in daily oral hygiene, within an interdisciplinary approach to the overall health of older adults living with AD [5].

#### 3.1.2 Dental and Root Caries

Dental caries in older adults, particularly those affected by AD, exhibits an epidemiological and clinical pattern distinct from that observed in younger populations. Whereas coronal caries activity tends to stabilize in most adults, older individuals experience a “third peak” of cariogenic activity [31], predominantly affecting exposed root surfaces due to gingival recession and periodontal attachment loss.

In individuals with AD, caries should therefore not be regarded solely as an isolated oral condition, but rather as an indirect marker of functional, cognitive, and systemic decline [31, 32]. Observational studies demonstrate that these individuals present a significantly higher mean number of decayed surfaces than cognitively preserved older adults, a difference that is particularly pronounced for root caries [33, 34]. Consistently, systematic and narrative reviews report a significantly higher prevalence of root caries in people with dementia, especially AD, compared with cognitively intact controls [31, 32].

The pathogenesis of root caries in AD is driven by qualitative and quantitative alterations in the oral microbiota, characterized by a disease‐specific dysbiotic state. The composition of the dental biofilm in older adults with AD differs substantially from that observed in healthy older individuals, with a marked increase in the relative abundance of acidogenic and aciduric microorganisms, particularly those belonging to the order *Lactobacillales* and the family *Streptococcaceae*—alongside a reduction in overall microbial diversity and a decline in genera associated with ecological homeostasis [34]. These microbial shifts promote a persistently acidic oral microenvironment, which favors the rapid progression of caries [35].

From a structural perspective, the root surface, composed of cementum and dentin, is inherently more susceptible to acid‐induced demineralization than enamel. The critical pH for dentin dissolution is substantially higher (approximately 6.2–6.7) than that of enamel (around 5.5) [36]. Therefore, in an oral environment characterized by reduced salivary buffering capacity, a frequent finding in individuals with AD due to polypharmacy [37] and salivary gland hypofunction [38, 39, 40], combined with ineffective mechanical biofilm removal, the progression of root caries tends to be accelerated [39]. Clinically, these lesions often present a circumferential pattern, leading to fracture of the clinical crown and retention of infected residual roots, which may act as chronic foci of infection and inflammation [41, 42, 43].

#### 3.1.3 Xerostomia and Hyposalivation

Saliva is a highly complex biological fluid that plays a pivotal role in maintaining oral homeostasis through its functions of lubrication, initial digestion, antimicrobial defense, buffering capacity, and dental remineralization [44, 45]. In the context of AD, significant alterations are observed in both salivary flow and qualitative composition [40, 46], with direct repercussions on the integrity of oral tissues and the ecological balance of the oral cavity.

Reduced salivary flow, characterizing hyposalivation, is a frequent finding in individuals with AD and, although often attributed to medication use, cannot be explained solely by this factor. Evidence suggests that even in the early stages of the disease, and after controlling for pharmacological variables, autonomic nervous system dysfunction occurs, affecting both sympathetic and parasympathetic innervation of major and minor salivary glands [40]. This neurofunctional impairment compromises basal and stimulated salivary secretion, contributing to the early onset of clinically relevant xerostomia [40].

A direct and clinically significant consequence of hyposalivation is the reduction in salivary buffering capacity. In patients with AD, reduced salivary flow results in delayed clearance and neutralization of exogenous or bacterially derived acids. Under acid challenge conditions, the impaired buffering and clearance capacity may allow oral pH to remain at extremely low levels for prolonged periods, falling well below the critical pH for enamel and dentin demineralization [47]. This chronically acidic oral environment not only accelerates the development of coronal and root caries but also favors dental erosion and colonization by aciduric and opportunistic microorganisms, such as *Candida albicans* [48, 49].

In addition, pharmacotherapy used in the management of AD exerts a substantial impact on salivary flow [50]. Acetylcholinesterase inhibitors, such as donepezil and rivastigmine, constitute first‐line symptomatic therapy aimed at increasing synaptic acetylcholine availability and temporarily preserving cognitive function [51]. However, in older adults with AD, this cholinergic effect rarely translates into improved salivary secretion, as these agents are commonly prescribed in combination with antidepressants, antipsychotics, and other medications with anticholinergic or xerostomic properties [52]. The cumulative effect of polypharmacy ultimately results in clinically significant hyposalivation.

#### 3.1.4 Traumatic Mucosal Lesions

The use of removable dentures in individuals with AD represents a relevant clinical challenge and is associated with a higher prevalence of mucosal lesions, particularly denture stomatitis [39, 53, 54]. This chronic inflammatory condition of the denture‐bearing mucosa, frequently associated with colonization by *Candida albicans*, occurs at a rate more than twice as high in patients with dementia compared with cognitively healthy older adults [55].

The etiology of denture‐related trauma is multifactorial and includes anatomical changes resulting from weight loss and progressive muscle atrophy during the course of AD, which compromise the fit of previously adequate prostheses [56]. The resulting denture instability promotes continuous mucosal friction and the development of traumatic ulcers. In advanced stages of the disease, the inability to report pain or discomfort may lead to prolonged use of ill‐fitting dentures, including those with metallic components, causing extensive soft tissue injury [54].

Poor denture hygiene and continuous denture wearing constitute major risk factors. The inability to remove dentures at night creates an anaerobic and acidified microenvironment beneath the denture base, favoring microbial and fungal proliferation [55]. Beyond local effects, these lesions may act as reservoirs of pathogens with aspiration potential, thereby increasing the risk of respiratory infections [57, 58]. Management is further complicated by delayed wound healing in older adults. Evidence suggests that denture adjustment combined with topical application of hyaluronic acid gel (0.2–0.3%) accelerates tissue repair compared to denture adjustment alone [59].

### 3.2 Clinical Strategies for Dental Management

A frequent error in the dental management of patients with dementia is the application of conventional treatment plans without adequate consideration of the stage of the disease. The literature suggests that treatment planning should consider the patient's functional capacity to tolerate procedures and to maintain oral hygiene, in addition to the technical necessity of the intervention [5, 60, 61]. As AD progresses, increasing cognitive and motor impairments emerge, requiring a gradual adaptation of the clinical approach, with a reduction in complex restorative procedures and a greater emphasis on preventive strategies and, in advanced stages, palliative care [5, 62].

Oral health professionals should individualize care by considering patient‐specific risks, prioritizing morning or early afternoon appointments, avoiding mealtimes, and ensuring a calm environment to minimize stress. Appointment duration should be short and efficient to reduce patient burden [5]. Table 1 summarizes dental management strategies according to the stage of the disease.

**TABLE 1:** Dental management strategies according to the stage of Alzheimer's disease.

Stage	Behavioral profile	Clinical guidance	Treatment objective	Dental planning	Recommended strategy
Early	Functional independence or need for minimal assistance. Preserved communication. Capacity to provide informed consent.	Patient‐centered anamnesis, validated by a caregiver when necessary. Appointments of up to 50 min.	Restore oral function and eliminate infectious foci. Plan for future functional decline.	Conventional outpatient dental care. Preventive and curative emphasis.	Conventional dental treatment. Oral hygiene education and training for patients, caregivers, and family members. Clinical interventions should be prioritized at this stage.
Moderate	Partial loss of autonomy. Temporal and spatial disorientation. Anxiety or resistance to dental care.	Mandatory caregiver presence. Short appointments (≈ 30 min), preferably in the morning. Consider home‐based dental care.	Maintain oral health and prevent pain.	Focus on maintenance of oral health and pain prevention.	Minimally invasive techniques (ART). Use of fluoride‐releasing materials. Possible conscious sedation. Intensive caregiver training for oral hygiene.
Advanced	Total dependence. Absent or limited verbal communication. Dysphagia and high aspiration risk.	Communication directed toward the patient to maintain engagement, with information obtained from caregivers. Very brief sessions focused on comfort.	Palliation, comfort, and prevention of systemic infection.	Multidisciplinary planning. Interventions restricted to pain and infection control.	Emergency treatment only. Assisted and palliative oral hygiene. Removal of dentures when there is risk. Use of cariostatic agents (e.g., SDF) for non‐restorative caries control.

Abbreviations: ART, atraumatic restorative treatment; SD, silver diamine fluoride.

After the diagnosis of AD, family members and/or caregivers should be promptly advised to schedule a dental evaluation, ideally as part of the initial recommendations provided by the medical team. The dentist should perform a comprehensive clinical examination to establish an individualized diagnosis and treatment plan, considering the progressive and irreversible nature of the disease [5, 15, 60, 61, 62].

### 3.3 Role of Caregivers in Oral Health Care

Caregivers play a central role in maintaining oral health in patients with AD, particularly as cognitive and functional decline progressively compromises the patient's ability to perform self‐care [4, 17]. This role encompasses both formal caregivers (health professionals or trained aides) and informal caregivers, especially family members, who often provide daily assistance and decision‐making support [4, 5].

As AD progresses, routine oral hygiene tasks such as toothbrushing, interdental cleaning, and denture care become increasingly dependent on caregivers. In moderate and advanced stages, patients often lose the ability to understand instructions, recognize the importance of oral hygiene, or cooperate with care, making caregiver involvement indispensable [4, 5, 60]. Inadequate assistance during this phase is strongly associated with increased plaque accumulation, periodontal disease, caries, mucosal lesions, and oral infections [41].

Beyond mechanical plaque control, caregivers are responsible for monitoring oral symptoms that patients may no longer be able to report, such as pain, bleeding, halitosis, mucosal ulcers, or ill‐fitting dentures [39]. Early identification of these signs is critical to prevent the progression of local oral conditions into systemic complications, including aspiration pneumonia, which represents a major cause of morbidity and mortality in individuals with advanced dementia [57, 58].

Caregivers also play a crucial role in denture management, ensuring the daily removal, cleaning, and proper storage of removable prostheses [63]. Continuous denture use, commonly observed in patients with cognitive impairment, significantly increases the risk of denture stomatitis, fungal infections, and traumatic mucosal lesions [55, 64, 65]. Education regarding nighttime denture removal and regular oral inspection is, therefore, essential [55, 63, 64, 65, 66].

However, caregivers frequently report a lack of training, limited knowledge, and emotional burden [67, 68, 69], which negatively impact oral care delivery. Many family caregivers prioritize general medical needs and feeding assistance, often underestimating the importance of oral health [70, 71]. Structured educational programs, practical demonstrations, and simplified oral care protocols have been shown to improve caregiver confidence, adherence, and patient outcomes [72, 73].

From a clinical perspective, the dentist must actively integrate caregivers into the treatment plan [73, 74]. This includes providing clear, stage‐appropriate instructions, selecting simplified hygiene aids (such as electric toothbrushes and high‐fluoride toothpaste), and tailoring preventive strategies to the caregiver's capacity and daily routine [75, 76]. Continuous reinforcement and follow‐up are fundamental, as caregiver turnover and disease progression are common.

In advanced stages of AD, caregivers become the primary agents of palliative oral care, focusing on comfort, infection control, and quality of life rather than restorative outcomes. At this stage, close communication between dental professionals, caregivers, and the multidisciplinary healthcare team is essential to ensure safe, ethical, and patient‐centered oral health management [74].

#### 3.3.1 Caregiver‐Oriented Oral Health Strategies

**FIGURE 2:**
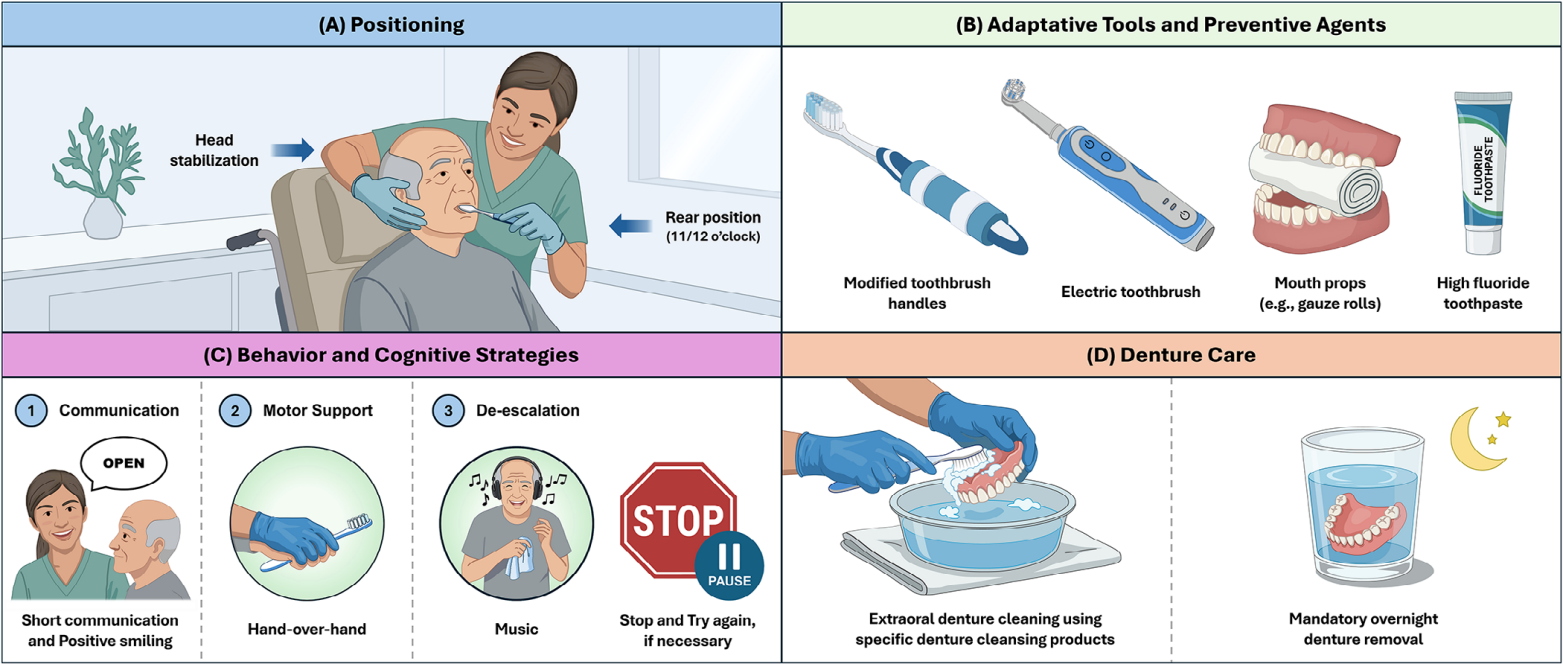
Caregiver‐oriented oral health strategies for older adults with Alzheimer's disease. (A) Safe positioning and head stabilization. (B) Use of adaptive hygiene tools and preventive agents. (C) Behavioral strategies (short communication, hand‐over‐hand guidance, music, and de‐escalation). (D) Denture care with daily cleaning and mandatory overnight removal.

In addition to professional guidance, the literature synthesized in this review supports a set of caregiver‐centered behavioral strategies designed to overcome cognitive deficits (particularly apraxia, agnosia, and impaired executive function) while leveraging preserved procedural memory, emotional memory, and sensory processing in individuals with AD. These strategies, illustrated in Figure 2, are consistently associated with improved cooperation, reduced resistance to care, and enhanced safety during oral hygiene procedures.

Positioning and physical stabilization (Figure 2A) represent a fundamental prerequisite for effective intervention. Proper caregiver positioning (11–12 o'clock) combined with gentle head support improves visual control and reduces unpredictable movements, facilitating the application of behavioral techniques and minimizing perceived threat, especially in patients with postural instability or advanced cognitive impairment.

Among behavioral strategies, short communication combined with positive affect (Figure 2C.1) is consistently emphasized. The evidence indicates that brief, concrete, single‐step verbal commands—delivered slowly, with calm tone, direct eye contact, and positive facial expression—are significantly more effective than complex explanations [77, 78]. As language comprehension deteriorates with disease progression, excessive verbal input increases confusion and resistance. Short, affirmative phrases (e.g., “Open,” “Good,” “All done”) reduce cognitive load and enhance task compliance, particularly in moderate and advanced stages of AD [77, 78, 79].

The hand‐over‐hand technique (Figure 2C.2) is one of the most robustly supported interventions for patients with apraxia or resistance to oral manipulation [78]. By guiding the patient's hand while holding the toothbrush or denture, the caregiver provides continuous proprioceptive feedback that preserves the sense of agency and activates residual procedural memory [80, 81]. Neurocognitively, this approach reduces the perception of external intrusion and promotes motor engagement [80, 81, 82]. The literature highlights its particular effectiveness during denture removal and insertion, as well as for initiating brushing movements in patients unable to start the task independently but capable of sustaining it once initiated [77, 78, 79].

Motor support strategies, including hand‐over‐hand guidance and mirrored movements, are further complemented by chaining and priming techniques, which reduce the cognitive demand of task initiation [78]. Initiating brushing or denture handling and subsequently transferring control to the patient activates automated motor sequences that remain relatively preserved in early to moderate dementia [77, 79]. Sensory priming, such as placing the toothbrush in the patient's hand, exposing them to the sound of running water, or allowing them to smell toothpaste, prepares relevant neural networks before motor execution, improving task acceptance [78, 80, 81].

For patients with sensory defensiveness or attentional instability, music‐based distraction (Figure 2C) emerges as an effective non‐pharmacological adjunct [78]. Familiar and emotionally salient music has been shown to modulate anxiety, reduce psychomotor agitation, and enhance cooperation during personal care activities [77]. By engaging preserved emotional and auditory memory pathways, music redirects attention away from the invasive nature of oral hygiene and facilitates a calmer behavioral state [77, 78]. Its effectiveness is greatest when individualized according to the patient's musical preferences and applied consistently during care routines.

When resistance escalates despite preventive strategies, rescuing and de‐escalation techniques (Figure 2C.3) are strongly supported. Immediate interruption of care, followed by either caregiver substitution or a temporal pause, prevents escalation from verbal refusal to physical aggression [77, 78]. This approach acknowledges the limited stress tolerance in AD and avoids reinforcing negative behavioral associations with oral care.

The effectiveness of these behavioral strategies is enhanced by the use of adaptive tools and preventive agents (Figure 2B), including modified toothbrush handles, electric toothbrushes (when tolerated), and high‐fluoride toothpaste [75, 76, 83, 84]. However, sensory hypersensitivity to vibration or noise requires individualized assessment [85, 86]. Mouth props, when correctly used, improve safety and access in patients with trismus or tonic bite reflex, reducing caregiver injury risk [83].

Finally, denture care strategies (Figure 2D) remain a core component of caregiver‐mediated oral health. Daily extraoral denture cleaning with specific denture cleansing products [87, 88] and mandatory overnight denture removal are consistently associated with reduced denture stomatitis, fungal colonization, and traumatic mucosal lesions [55, 64, 65]. Combining denture care with behavioral strategies (particularly hand‐over‐hand guidance and short communication) further improves acceptance and adherence.

## 4 Conclusion and Future Directions

Although further longitudinal studies are needed to clarify mechanistic links between oral inflammation, neuroinflammation, and cognitive decline, current evidence supports immediate clinical translation. Individuals with AD experience progressive oral health deterioration, with relevant consequences for comfort, nutrition, infection control, and quality of life.

Prevention remains central to oral care in this population, as cognitive and functional decline limits the feasibility of complex interventions. Dental management should be stage‐specific and guided by functional capacity, prioritizing simplified preventive strategies early and transitioning toward palliative, comfort‐oriented care as the disease advances. Oral health deterioration reflects the combined effects of functional impairment, salivary dysfunction, and barriers to care.

Early integration of dental assessments into routine medical care at AD diagnosis may facilitate timely preventive and supportive interventions. In this context, teledentistry represents a useful adjunct by enabling remote triage, reducing unnecessary transportation, and supporting caregivers through education and guidance on daily oral hygiene. Ultimately, embedding dementia‐informed principles into dental practice and interdisciplinary care models is essential to preserve dignity and improve quality of life in individuals living with AD.

## Author Contributions

**Mayron Guedes Silva**: writing – original draft, methodology, investigation, data curation, conceptualization, visualization, resources, project administration, funding acquisition. **Matheus Bastos Vasconcelos**: writing – original draft, methodology, investigation, data curation, visualization, funding acquisition. **Raphael Guedes Silva**: Writing – review & editing, Visualization, Funding acquisition. **Maria Áurea Lira Feitosa**: Writing – review & editing, Visualization, Validation, Supervision, Project administration.

## Funding

This study was financed by the Coordenação de Aperfeiçoamento de Pessoal de Nível Superior—Brasil (CAPES)—Finance Code 001.

## Conflicts of Interest

The authors declare no conflicts of interest.
